# Structural and biochemical studies of the distinct activity profiles of Rai1 enzymes

**DOI:** 10.1093/nar/gkv620

**Published:** 2015-06-22

**Authors:** Vivien Ya-Fan Wang, Xinfu Jiao, Megerditch Kiledjian, Liang Tong

**Affiliations:** 1 Department of Biological Sciences, Columbia University, New York, NY 10027, USA; 2 Department of Cell Biology and Neuroscience, Rutgers University, Piscataway, NJ 08854, USA

## Abstract

Recent studies showed that Rai1 and its homologs are a crucial component of the mRNA 5′-end capping quality control mechanism. They can possess RNA 5′-end pyrophosphohydrolase (PPH), decapping, and 5′-3′ exonuclease (toward 5′ monophosphate RNA) activities, which help to degrade mRNAs with incomplete 5′-end capping. A single active site in the enzyme supports these apparently distinct activities. However, each Rai1 protein studied so far has a unique set of activities, and the molecular basis for these differences are not known. Here, we have characterized the highly diverse activity profiles of Rai1 homologs from a collection of fungal organisms and identified a new activity for these enzymes, 5′-end triphosphonucleotide hydrolase (TPH) instead of PPH activity. Crystal structures of two of these enzymes bound to RNA oligonucleotides reveal differences in the RNA binding modes. Structure-based mutations of these enzymes, changing residues that contact the RNA but are poorly conserved, have substantial effects on their activity, providing a framework to begin to understand the molecular basis for the different activity profiles.

## INTRODUCTION

The 5′-end m^7^GpppN cap, which is formed co-transcriptionally on pre-mRNAs, is important for subsequent steps in gene expression, including mRNA splicing, polyadenylation, nuclear export, stability and translation efficiency (1–5). Cap formation is catalyzed by the sequential actions of RNA 5′ triphosphatase, guanylyltransferase and methyltransferase (6). First, the 5′ triphosphate of the primary transcript from RNA polymerase II (Pol II) is converted to diphosphate. Then, a cap is formed by the attachment of GMP in a 5′-5′ pyrophosphate linkage. Finally, a mature cap is produced by the methylation of the guanine at the N^7^ position.

Decapping is associated with mRNA turnover, decay, and quality control (7–12). It is a highly regulated process and is catalyzed by the decapping enzymes Dcp2 and Nudt16, releasing 7-methylguanosine diphosphate (m^7^Gpp) and 5′ monophosphate RNA. Several other members of the Nudix family also possess decapping activity (13). Following decapping, the 5′ monophosphate RNA is rapidly degraded by the cytoplasmic 5′-3′ exoribonuclease Xrn1 (14,15).

We recently discovered a quality surveillance mechanism for mRNA 5′-end capping (16–20), in contrast to the general belief that capping always proceeds to completion and no quality control is necessary. We found that *Schizosaccharomyces pombe* Rai1, the protein partner of the nuclear 5′-3′ exonuclease Rat1 (21,22), possesses RNA 5′ pyrophosphohydrolase (PPH) activity, releasing pyrophosphate (PP_i_) from 5′ triphosphate RNA (such as the primary Pol II transcript) (16). Rai1 also possesses a novel decapping activity, with strong preference for unmethylated cap and releasing GpppN (17), in contrast to the classical decapping enzymes. These biochemical activities would enable the degradation of 5′-end capping intermediates, as they would otherwise be protected against Xrn1, Dcp2 and Nudt16 (16). We subsequently demonstrated the existence of incompletely capped RNAs in yeast and mammalian cells, and that Rai1 proteins have central roles in facilitating the degradation of these RNAs (17–19).

**Figure 1.**
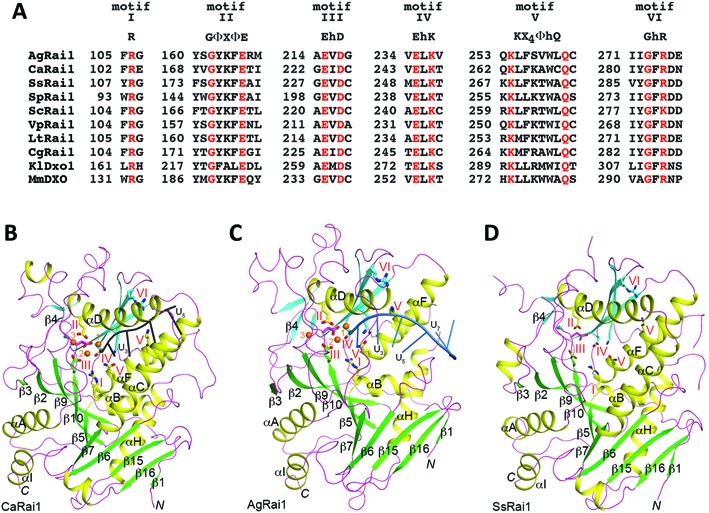
Conserved sequence motifs among Rai1/Dxo1/DXO homologs. (**A**) Alignment of residues near the six conserved motifs in representative sequences of Rai1/Dxo1/DXO homologs, including several fungal Rai1 homologs, *K. lactis* Dxo1 (KlDxo1) and mouse DXO (MmDXO). (**B**) Overall structure of *Candida albicans* Rai1 (CaRai1) in a ternary complex with the pU5 RNA oligo (black) and Mn^2+^ (orange spheres) at 2.0 Å resolution. The two β-sheets in the structure are shown in green and cyan. The side chains of the six conserved motifs are shown as stick models and labeled. (**C**) Overall structure of *A. gossypii* Rai1 (AgRai1) in a ternary complex with the pU(S)6 RNA oligo (light blue) and Mn^2+^ (orange spheres) at 2.4 Å resolution. (**D**) Overall structure of *S. stipitis* Rai1 (SsRai1) at 1.64 Å resolution. The three structures are reported in this paper. All the structure figures were produced with PyMOL (www.pymol.org).

Rai1 homologs share several highly conserved sequence motifs (18) (Figure 1a), and our structural studies show that they are located in the active site region, mediating the binding of the divalent metal ions and/or the RNA (Figure 1b–d) (16,18,19). These motifs include an Arg residue (motif I), GΦXΦE (motif II, where Φ is an aromatic or hydrophobic residue and X any residue), EhD (motif III, where h is a hydrophobic residue), EhK (motif IV), KX_4_ΦhQ (motif V), and GhR (motif VI, GhK in yeast Rai1). On the other hand, the amino acid sequences outside of these motifs are generally poorly conserved among these proteins (Supplementary Figure S1). For example, *S. pombe* Rai1 has 26% overall sequence identity with mouse DXO (19), a mammalian homolog.

Moreover, the three Rai1 homologs that have been characterized biochemically, *S. pombe* Rai1 (SpRai1) (16,17), *Kluyveromyces lactis* Dxo1 (Ydr370C, KlDxo1) (18) and mouse DXO (Dom3Z, MmDXO) (19), show differing activity profiles (Table 1). SpRai1 has PPH activity and decapping activity toward unmethylated caps. KlDxo1 has decapping activity toward both methylated and unmethylated caps, but no PPH activity. KlDxo1 also has distributive, non-processive 5′-3′ exoribonuclease activity. MmDXO has PPH activity, decapping activity that is nonselective regarding the methylation status of the cap, and distributive 5′-3′ exonuclease activity. Our structures of MmDXO in complex with RNA indicate that the same catalytic machinery mediates all of these activities, and it is the differing binding modes of the substrates that define the outcome of the reaction (19). However, the molecular basis for why each Rai1 protein appears to have a unique set of catalytic activities is not well understood.

**Table 1.** Summary of catalytic activities of Rai1 homologs

Activity	PPH	5′ Triphospho-nucleotide hydrolase (TPH)	Decapping		5′-3′ Exoribonuclease
Substrate	pppRNA	pppRNA	GpppRNA	m^7^GpppRNA	pRNA
Products	PP_i_ + pRNA	pppN + pRNA	GpppN + pRNA	m^7^GpppN + pRNA	pN + pRNA
AgRai1	–	++	++	++	++
CaRai1	–	–	+	–	–
CgRai1	–	+	+	–	–
LtRai1	–	–	+/–	–	+/–
ScRai1	–	–	+/–	–	–
SpRai1	+	–	+	–	–
SsRai1	–	+/–	+	+/–	+
VpRai1	–	–	+	–	–
KlDxo1^a^	–	–	++	++	++
MmDXO^b^	++	–	++	++	++

+/–, ≤10% activity; +, ≤50% activity; ++, ≥50% activity.

^a^ Data from (18).

^b^ Data from (19).

In this study, we have characterized the catalytic activity of Rai1 homologs from a collection of fungal species and determined the high-resolution crystal structures for three of them, two of them in complex with RNA oligonucleotides. We have found substantial variations in the activity profiles of the Rai1 homologs, and discovered that some of them possess a new 5′ triphosphonucleotide hydrolase (TPH) activity. The binding modes of the first two nucleotides of the RNA body are similar among the Rai1 homologs, and generally involve conserved residues. On the other hand, large differences are observed in the binding modes of the rest of the RNA. Mutations of residues that contact the RNA but are poorly conserved among the Rai1 homologs had significant impacts on the activity of the enzymes, suggesting that they play important roles in determining the activity profile of each enzyme.

## MATERIALS AND METHODS

### Protein expression, purification, and crystallization

Full-length *Ashbya gossypii* (Ag), *Scheffersomyces stipitis* (Ss), *Candida albicans* (Ca), *Candida glabrata* (Cg), *Lachancea thermotolerans* (Lt), *S. cerevisiae* (Sc), *S. pombe* (Sp) and *Vanderwaltozyma polyspora* (Vp) Rai1 were cloned into the pET28a (N-terminal His tag) and pET26b (C-terminal His tag) vectors (Novagen). The proteins were overexpressed in *Escherichia coli*. BL21 Star (DE3) cells at 16°C and purified by Ni-NTA Superflow (Qiagen) and gel filtration (Sephacryl S-300, GE Healthcare) chromatography. The proteins were concentrated to 25–30 mg/ml in a buffer containing 20 mM Tris (pH 7.5), 250 mM NaCl and 5% (v/v) glycerol, frozen in liquid nitrogen and stored at –80°C.

CaRai1 crystals were obtained by the sitting-drop vapor diffusion method at 20°C with a reservoir solution containing 0.1 M Bis–Tris (pH 5.5) and 18% (w/v) PEG 3350. The CaRai1–pU5–Mn^2+^ complex crystals were obtained by using 1:2 protein:RNA molar ratio, and 10 mM MnCl_2_, in the same condition as the CaRai1 free enzyme crystals.

AgRai1 crystals were obtained by the sitting-drop vapor diffusion method at 20°C with a reservoir solution containing 0.1 M phosphate-citrate (pH 4.2), 20% (w/v) PEG 300, 0.2 M (NH_4_)_2_SO_4_ and 10% (v/v) glycerol. The AgRai1–pU(S)6–Mn^2+^ complex was obtained by soaking the free AgRai1 crystals with 10 mM pU(S)6 RNA and 10 mM MnCl_2_ overnight at 20°C in the presence of 20% (w/v) PEG 3350 and 10% (v/v) ethylene glycol.

SsRai1 crystals were obtained by the sitting-drop vapor diffusion method at 20°C with a reservoir solution containing 20% (w/v) PEG 3350 and 200 mM NH_4_Cl. All crystals were flash frozen in liquid nitrogen for diffraction screening and data collection at 100 K.

### Data collection and structure determination

X-ray diffraction data were collected at the National Synchrotron Light Source (NSLS) beamline X29A. The diffraction images were processed and scaled using the HKL package (23). The structures were solved with the molecular replacement method with the program Phaser (24) and the structure of SpRai1 (16) as the search model. The structure refinement was carried out with the Crystallography and NMR System (CNS) (25), Refmac (26) and PHENIX (27). The atomic models were built with the Coot program (28). The crystallographic information is summarized in Table 2.

**Table 2.** Summary of crystallographic information

Structure	AgRai1	CaRai1	SsRai1	AgRai1–pU(S)6–Mn^2+^	CaRai1–pU5–Mn^2+^
Data collection					
Space group	*C*222_1_	*C*222_1_	*P*2_1_	*P*2_1_2_1_2_1_	*P*2_1_2_1_2
Cell dimensions					
*a, b, c* (Å)	79.0, 164.2, 66.1	85.2, 98.8, 115.6	52.9, 104.5, 78.7	69.6, 79.7, 160.1	97.8, 114.6, 84.4
α, β,γ (°)	90, 90, 90	90, 90, 90	90, 98.1, 90	90, 90, 90	90, 90, 90
Resolution (Å)^a^	50–1.8 (1.86–1.8)	50–2.2 (2.28–2.2)	50–1.64 (1.7–1.64)	50–2.4 (2.5–2.4)	50–2.0 (2.07–2.0)
*R*_merge_ (%)	6.2 (35.5)	5.6 (22.7)	3.6 (23.8)	12.4 (46.5)	9.0 (42.2)
*I*/*σI*	24.0 (4.7)	27.4 (6.2)	29.7 (5.4)	15.3 (3.6)	19.6 (4.2)
Completeness (%)	100 (100)	97 (95)	99 (96)	100 (99)	100 (99)
Redundancy	5.6 (5.6)	5.9 (4.6)	3.7 (3.5)	6.1 (5.5)	6.9 (6.5)
Refinement					
Resolution (Å)	50–1.8	50–2.2	50–1.64	50–2.4	50–2.0
No. of reflections	38 099	23 150	100 257	33 896	61 906
*R*_work_/*R*_free_ (%)	18.7/23.0	22.9/29.2	16.3/21.7	19.6/25.4	18.6/24.3
No. of atoms					
Protein	2891	3218	6169	5935	6407
RNA	–	–	–	242	166
Ions	25	–	–	16	6
Water	300	78	849	357	1,044
B-factors					
Protein	25.2	56.0	29.6	27.5	32.2
RNA	–	­–	–	21.7	42.6
Ions	28.8	–	–	21.7	39.1
Water	36.5	51.6	43.1	26.7	47.9
R.m.s. deviations					
Bond lengths (Å)	0.010	0.015	0.006	0.012	0.010
Bond angles (°)	1.3	1.7	0.9	1.6	1.4

^a^The numbers in parentheses are for the highest resolution shell. One crystal was used for each data collection.

### Mutagenesis and activity assays

Structure-based site-specific mutations were generated following the QuikChange Kit (Stratagene) and sequenced for verification of the correct incorporation of the target mutations.

The 5′-end-cap ^32^P-labeled RNAs were generated with Vaccinia virus mRNA capping enzyme in the presence of [α-^32^P]GTP with or without S-adenosyl-methionine (SAM) as descripted previously (29,30). The 5′-end ^32^P-labeled and uniformly^32^P-labeled triphosphate RNAs were generated by *in vitro* transcription from pcDNA3 polylinker PCR DNA template with T7 RNA polymerase in the presence of [γ-^32^P]GTP or [α-^32^P]GTP. To generate uniformly ^32^P-labeled RNAs, the cap analogs, GpppG or m^7^GpppG, were added to the transcription reactions to produce non-methylated or a mixture of 5′-end methylated and non-methylated capped RNAs, respectively. 5′-end methylation was further enhanced by subjecting the latter RNA to Vaccinia virus capping enzyme and SAM, as described above, to generate m^7^G-capped RNA. Uniformly^32^P-labeled RNA with a 5′-end hydroxyl was generated by treating uniformly^32^P-labeled triphosphate RNA with calf intestinal alkaline phosphatase (CIP).

Exoribonuclease assays in Figures 3a, c and 6c with ^32^P-labeled RNA were performed as previously reported (18,19). Briefly, assays were carried out in 20 μl reactions in RNA decay buffer (10 mM Tris (pH 7.5), 100 mM NaCl, 2 mM MgCl_2_, 1 mM DTT), 1 mM MnCl_2_, 20 U RNasin RNase inhibitor (Promega), 5′-end radio-labeled RNA (∼100 counts per second) and 100 nM of the indicated histidine-tagged Rai1 proteins. Reactions were stopped with 40 μl stop buffer (50% formamide, 5 M urea and 30 mM EDTA) and resolved by 7 M urea denaturing PAGE (5%). Dried gels were exposed to Phosphor Screen and ^32^P-labeled RNA detected by a Molecular Dynamics PhosphorImager (Storm860) and quantitated with Image Quant software.

**Figure 2.**
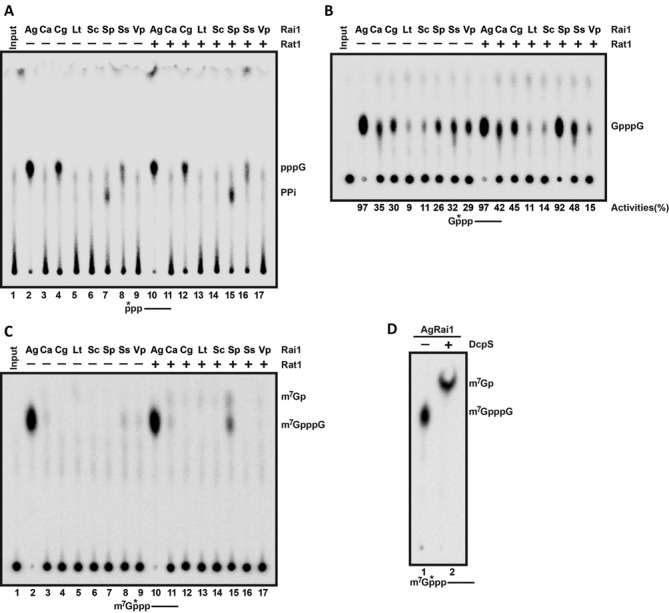
Pyrophosphohydrolase (PPH) and decapping activities of fungal Rai1 homologs. (**A**) Activity of Rai1 homologs toward 5′ triphosphate RNA, in the absence or presence of SpRat1. A schematic of the RNA used as the substrate is indicated at the bottom, with the asterisks denoting the position of the ^32^P label. The reaction product is monitored by thin-layer chromatography (TLC). AgRai1, CgRai1 and SsRai1 have a novel activity, releasing GTP (pppG) rather than PPi. (**B**) The decapping activity toward unmethylated capped RNA. The percentage of substrate turnover is indicated at the bottom. (**C**) The decapping activity toward mature methylated capped RNA. (**D**) DcpS further hydrolyzed the m^7^GpppG product from AgRai1 to produce N^7^-methyl GMP (m^7^Gp).

**Figure 3.**
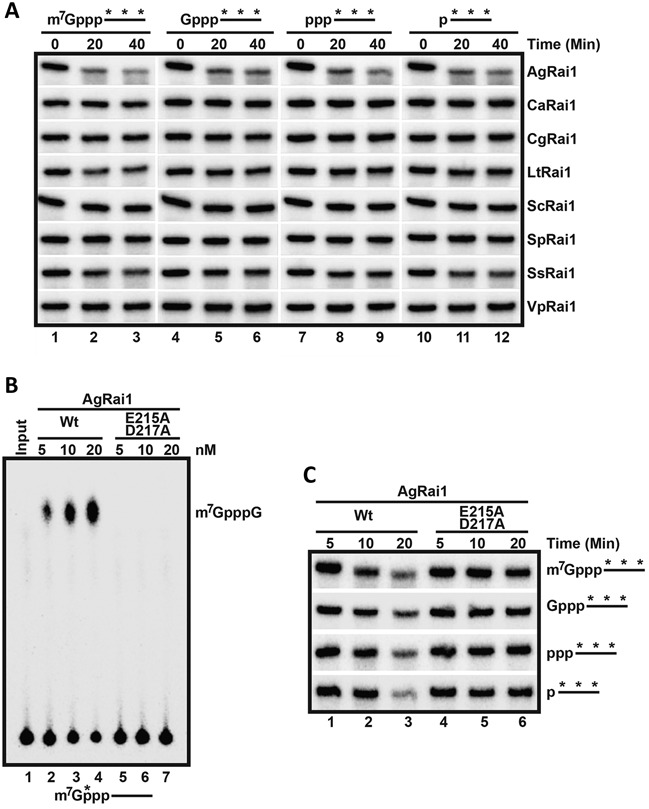
5′-3′ exoribonuclease activity of fungal Rai1 homologs. (**A**) Activity of Rai1 homologs toward body-labeled RNA with different 5′ modifications. (**B**) The E215A/D217A double mutant of AgRai1 abolished its decapping activity toward methylated capped RNA. (**C**) The double mutant also abolished the 5′-3′ exonuclease activity.

**Figure 4.**
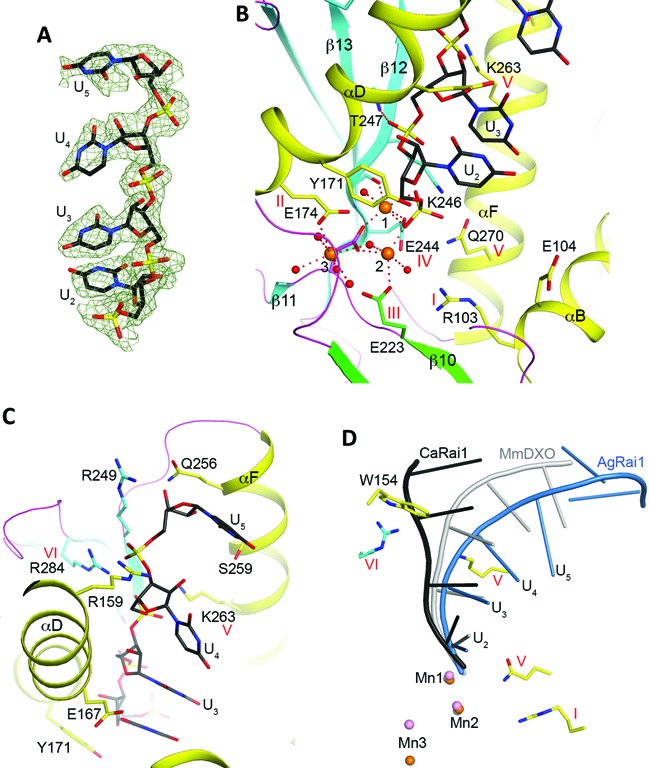
Detailed interactions between CaRai1 and the pU5 RNA. (**A**) 2*F*_o_ – *F*_c_ electron density for the pU5 RNA at 2.0 Å resolution, contoured at 1*σ*. (**B**) Interactions between the first two nucleotides of the pU5 RNA (U_2_ and U_3_) and CaRai1. The liganding interactions of the three Mn^2+^ ions are indicated with the dashed lines in red, and the water ligands are shown as red spheres. Side chains of amino acids are shown as stick models. (**C**) Interactions between U_4_ and U_5_ of the pU5 RNA and CaRai1. (**D**) Overlay of the binding modes of pU5 RNA to CaRai1 (black), pU(S)6 RNA to AgRai1 (light blue), and pU5 RNA to MmDXO (gray) (19). The Mn^2+^ ions are shown in orange (AgRai1), pink (CaRai1), and gray (MmDXO) and labeled. Selected amino acid side chains from AgRai1 are shown as stick models. The side chain of Trp154 clashes with the RNA in CaRai1 and MmDXO.

**Figure 5.**
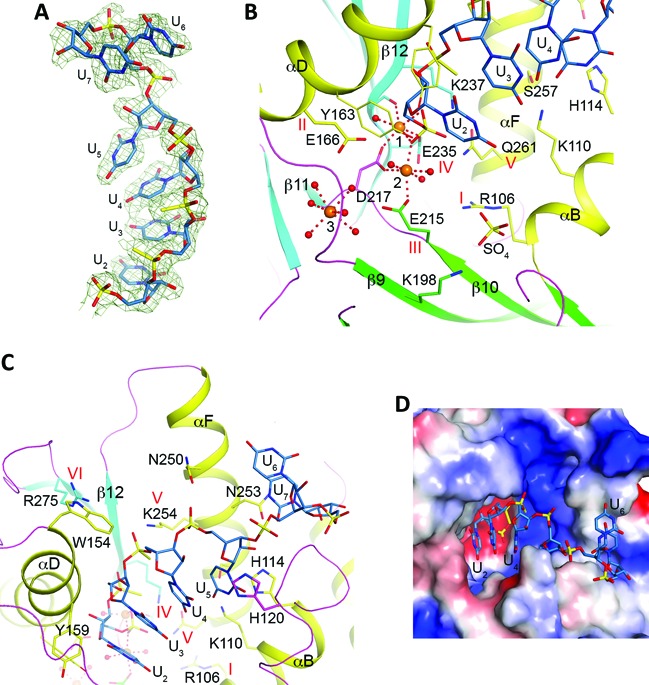
Detailed interactions between AgRai1 and the pU(S)6 RNA. (**A**) 2*F*_o_ – *F*_c_ electron density for the pU(S)6 RNA at 2.4 Å resolution, contoured at 1*σ*. (**B**) Interactions between the first two nucleotides of the pU(S)6 RNA (U_2_ and U_3_, light blue) and AgRai1. A sulfate ion bound in the active site region is shown as stick models. (**C**) Interactions between U_4_ through U_7_ of the pU(S)6 RNA and AgRai1. (**D**) Molecular surface of AgRai1 in the active site region, colored by electrostatic potential. The pU(S)6 RNA is shown as stick models. The last two nucleotides interact with the opening of the active site pocket.

**Figure 6.**
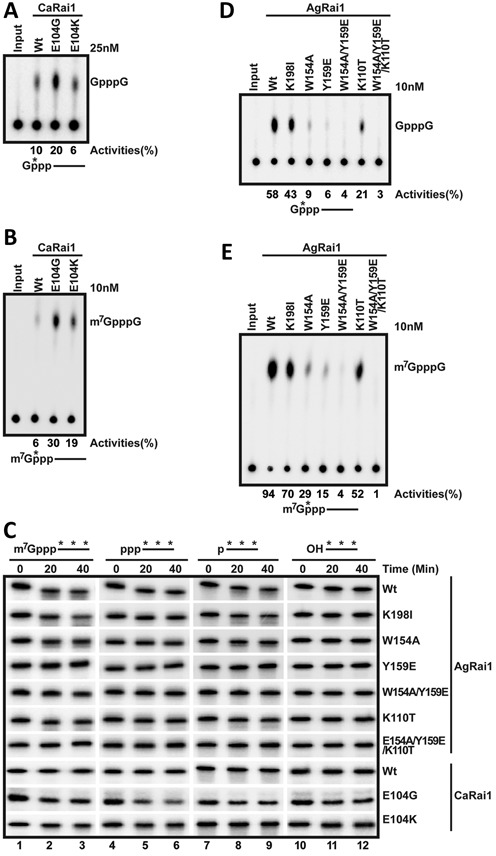
Structure-based mutations have great impacts on catalytic activity. (**A**) Decapping activity of the wild-type CaRai1 and the E104G and E104K mutants toward unmethylated, capped RNA. (**B**) Decapping activity of the wild-type CaRai1 and the E104G and E104K mutants toward methylated, capped RNA. (**C**) Activity of CaRai1 and AgRai1 wild-type enzymes and mutants toward body-labeled RNA with different 5′ modifications. (**D**) Decapping activity of the wild-type AgRai1 and various mutants toward unmethylated, capped RNA. (**E**) Decapping activity of the wild-type AgRai1 and various mutants toward methylated, capped RNA.

Decapping assays were carried out with the indicated ^32^P-labeled RNAs and 50 nM of the indicated protein for 30 min at 37°C analogous to the same buffer conditions as the exonuclease reactions above. Decapping products were resolved on polyethyleneimine (PEI)-cellulose thin-layer chromatography (TLC) plates developed in 0.45 M (NH_4_)_2_SO_4_ or 1.5 M K_2_SO_4_ (pH 3.4, Figure 2a) and visualized and quantitated on PhosphorImager as above.

For the PPH assay, A PPi marker generated enzymatically with bacterial RppH (31) was used. RppH will hydrolyze ^32^pppRNA to ^32^PPi and pRNA. Pi is generated by phosphatase treatment of the same RNA.

## RESULTS AND DISCUSSION

### Fungal Rai1 homologs have diverse activity profiles

To better characterize the range of activity profiles of Rai1 proteins, we expressed and purified full-length Rai1 homologs from a collection of fungal species, including *A. gossypii* (AgRai1), *C. albicans* (CaRai1), *C. glabrata* (CgRai1), *L. thermotolerans* (LtRai1), *S. cerevisiae* (ScRai1), *S. stipitis* (SsRai1) and *V. polyspora* (VpRai1). This subset of Rai1 proteins share stronger overall sequence conservation than other Rai1 proteins (Supplementary Figure S1). For example, ScRai1 and AgRai1 share 47% sequence identity, while AgRai1 and SsRai1 share 39% sequence identity. We also included SpRai1 in these experiments. The homologs of Dxo1, a weak sequence homolog of Rai1 found in a collection of fungal organisms, were studied earlier (18) and were not pursued here.

To study the PPH activity, a 5′ triphosphate RNA with a ^32^P label at the γ position was used as the substrate (Figure 2a). As we reported earlier (16), SpRai1 possessed PPH activity, releasing PP_i_ as a product (lane 7), and this activity was weakly stimulated by the presence of SpRat1 (lane 15). AgRai1, CgRai1 and SsRai1 also showed activity toward this substrate, with AgRai1 having the strongest activity among all the homologs. However, these enzymes released GTP (pppG) as a product rather than PP_i_ (Figure 2a, lanes 2, 4 and 8), demonstrating another catalytic activity for Rai1 proteins. Therefore, AgRai1, CgRai1 and SsRai1 have a distinct 5′ triphosphonucleotide hydrolase (TPH) activity and do not have PPH activity. SpRat1 did not appear to have any effect on this activity of the three enzymes.

All the Rai1 proteins tested showed decapping activity toward unmethylated capped RNA (GpppG-RNA), releasing the GpppG cap structure as a product (Figure 2b). AgRai1 again showed the highest activity; CaRai1, CgRai1, SpRai1, SsRai1 and VpRai1 showed moderate activity; and LtRai1 and ScRai1 are only slightly active under the condition tested. SpRat1 substantially stimulated the activity of SpRai1, but had only minor effects on the other Rai1 homologs (Figure 2b). It remains possible that the activity of a specific Rai1 protein can be stimulated to a greater extent by Rat1 from the same fungal species.

For mature, methylated capped RNA, AgRai1 showed strong activity, releasing m^7^GpppG as a product (Figure 2c), which can be further hydrolyzed by the human scavenger decapping enzyme DcpS to release N^7^ methylated GMP (m^7^Gp) (Figure 2d) (11). SpRai1 showed very weak activity toward this substrate in the presence of SpRat1, and might produce m^7^Gp as a product in addition to m^7^GpppG (Figure 2c). The other Rai1 enzymes showed essentially no activity toward this substrate.

Next, we tested the 5′-3′ exoribonuclease activity of the different Rai1 proteins, using a body-labeled 5′ monophosphate RNA as the substrate (Figure 3a, lanes 10–12). AgRai1 again showed strong activity, while consistently weaker activity was observed for SsRai1. The other proteins showed minimal if any activity in this assay. We also used body-labeled RNAs with other 5′-end modifications (triphosphate, unmethylated cap and methylated cap) as substrates, and the observed results on the 5′-end modified RNAs were generally consistent with those from the 5′ monophosphate RNA (Figure 3a, Supplementary Table S1). To confirm that the observed activity is truly due to AgRai1 rather than a contaminating protein, we produced the E215A/D217A double mutant, which should eliminate the binding of metal ions to the active site (16,19). The mutant protein displayed neither decapping (Figure 3b) nor 5′-3′ exonuclease activity toward 5′ monophosphate or triphosphate RNA (Figure 3c, Supplementary Table S2).

Overall, our biochemical characterizations of the fungal Rai1 homologs indicate that they have diverse activity profiles (Table 1), despite their substantial amino acid sequence conservation. Moreover, AgRai1, CgRai1 and SsRai1 have a unique 5′ triphosphonucleotide hydrolase (TPH) activity, releasing the first triphosphorylated nucleotide, rather than PPH activity (Figure 2a). The results also consistently show that AgRai1 has the strongest activities in the buffer conditions tested.

### Overall structures of AgRai1, CaRai1 and SsRai1 are similar to those of other Rai1 homologs

To help understand the molecular basis for the diverse activity profiles of the Rai1 enzymes, we next determined the crystal structures at high resolution (2.4–1.64 Å, Table 2) of wild-type AgRai1, CaRai1 and SsRai1 free enzymes, CaRai1 in a ternary complex with Mn^2+^ and an RNA penta-nucleotide with a 5′ monophosphate (pU5) (19), and AgRai1 in a ternary complex with Mn^2+^ and an RNA hexa-nucleotide with a 5′ monophosphate and with the phosphodiester group between nucleotides 1 and 2 and 2 and 3 replaced with a phosphorothioate group to inhibit hydrolysis (pU(S)6) (19). The CaRai1–pU5–Mn^2+^ complex was obtained by co-crystallization with pU5 RNA using 1:2 protein:RNA molar ratio in the presence of 10 mM MnCl_2_. The AgRai1–pU(S)6–Mn^2+^ complex was prepared by soaking AgRai1 free enzyme crystals with 10 mM pU(S)6 and 10 mM MnCl_2_ overnight. All the structures have good agreement with the X-ray diffraction data and the expected bond lengths, bond angles, and other geometric parameters (Table 2). There are essentially no conformational changes in CaRai1 upon RNA binding, with rms distance of 0.38 Å for the Cα atoms of the two structures. The rms distance is 0.62 Å for AgRai1, and small changes for helix αF (containing motif V) and other regions are visible (Supplementary Figure S2), although these changes are unlikely to seriously affect RNA binding. The structures of the AgRai1 and CaRai1 free enzymes will not be described further here.

The overall structures of CaRai1 (Figure 1b), AgRai1 (Figure 1c) and SsRai1 (Figure 1d) are similar to those of SpRai1 (16), KlDxo1 (18), and MmDXO (16,19). Each structure contains two mixed β-sheets that are surrounded by α-helices. The CaRai1 and SsRai1 structures are especially similar (Supplementary Figure S3), consistent with the 58% sequence identity between them. At the same time, there are substantial differences in the positions of the helices, the connecting loops and even some of the β-strands among some of the structures, and these differences are especially pronounced for the β4-αA loop, αC and αD helices and the connecting loop, which form one wall of the active site pocket. The αC helix is absent in AgRai1 but contains more than four turns in CaRai1 and SsRai1, which also have a longer αD helix. The residues in these segments are not well conserved among the Rai1 proteins (Supplementary Figure S1), and the surface of this wall is positioned near the bases of the RNA.

### Binding mode of pU5 in CaRai1

In the CaRai1–pU5–Mn^2+^ ternary complex, clear electron density was observed for the first four nucleotides of the RNA (Figure 4a). Two Mn^2+^ ions are coordinated by residues in motifs II (Glu174, αD), III (Glu223 and Asp225, β10 and β10–β11 loop) and IV (Glu244, β12) (Figure 4b), as observed earlier in the MmDXO–pU5–Mn^2+^ complex (19). Similarly, one of the terminal oxygen atoms of the 5′ monophosphate, the scissile phosphate of the substrate, is a bridging ligand to both metal ions. Therefore, the RNA is bound as a product of the reaction, and hence its nucleotides are numbered U_2_ through U_5_, while nucleotide U_1_ would be the leaving group of the substrate. Even through CaRai1 has relatively low activity in our assays (Table 1), it may be more active under other conditions, especially in the presence of CaRat1. This 5′ phosphate group is also located near residues in motifs I (Arg103, αB) and V (Gln270, αF) (Figure 4b), suggesting that these two residues may contribute to binding the 5′ segment of the substrate. The side chain of Tyr171 (part of motif II) is packed against the ribose of U_2_. A third Mn^2+^ ion is observed in the active site region, with the side chain of Glu174 (motif II) as its only ligand from the protein or RNA and one of its coordinating water molecules is shared with the second Mn^2+^ ion (Figure 4b). It is not clear whether this third metal ion has any role in catalysis.

The structure revealed that Glu104 (αB) is located near the expected binding site for the 5′ segment of the substrate (Figure 4b). The side chain has weak electron density and assumes different conformations in the two CaRai1 molecules in the asymmetric unit (Supplementary Figure S4). This residue is unique to CaRai1, and is a Gly in the other Rai1 proteins (Supplementary Figure S1). The presence of the negative charge and the larger size of the side chain could interfere with the binding of the substrate through electrostatic and steric repulsions.

The bases of the nucleotides are not specifically recognized, and their electron density is also weaker (Figure 4a). This is consistent with the biochemical data showing that Rai1 proteins do not have sequence specificity toward the RNA substrate, and the fact that the residues contacting the bases are not well conserved among Rai1 proteins. The base of U_3_ is stacked with that of U_2_, but this stacking interaction is not maintained by U_4_ and U_5_, and the RNA backbone is relatively straight in this complex (Figure 4c). The backbone phosphate group of U_3_ is hydrogen-bonded to the main-chain amide of Thr247 (in strand β12, the residue immediately following motif IV, Figure 4b). The phosphate of U_4_ has ionic interactions with the side chains of Lys263 (αF, motif V) and Arg284 (β13, motif VI), while that of U_5_ has ionic interactions with Arg284 (motif VI) and Arg159 (αD). Arg159 is not conserved among Rai1 homologs (Supplementary Figure S1), and the unique interaction with this residue may have helped define the bound conformation of pU5 to CaRai1. The ribose and base of U_5_ are placed near helix αF, and have weak interactions with a few of its side chains (Figure 4b).

There are two copies of the CaRai1–pU5–Mn^2+^ complex in the asymmetric unit of the crystal. The overall structures of the two protein molecules are essentially the same, with rms distance of 0.37 Å for their equivalent Cα atoms, and the binding modes of the two RNA molecules are nearly identical as well. One notable difference between the two complexes is that the side chain of Glu223 (motif III) in the second monomer assumes a different rotamer and is not coordinated to the second Mn^2+^ ion (Supplementary Figure S4).

Compared to the structure of the MmDXO–pU5–Mn^2+^ complex (19), the binding modes of the two metal ions and the first two nucleotides of the RNA (U_2_ and U_3_) are similar (Figure 4d). There is a noticeable difference in the position of U_4_, especially its base. The position of U_5_ in the CaRai1 complex is entirely different from that in the MmDXO complex, possibly due to the interaction with Arg159.

### Binding mode of pU(S)6 in AgRai1

Clear electron density was observed for all six nucleotides of the RNA in the AgRai1–pU(S)6–Mn^2+^ ternary complex (Figure 5a). The two copies of the complex in the asymmetric unit have essentially the same conformation, with rms distance of 0.14 Å for their equivalent Cα atoms. The structure reveals that pU(S)6 is also bound as a product to the active site of AgRai1, with its 5′ phosphate being a bridging ligand to both Mn^2+^ ions (Figure 5b), in contrast to our observations with MmDXO where the binding mode of pU(S)6 mimics the substrate (19). There is also a third Mn^2+^ near the active site, but its position is 3.3 Å from that observed in CaRai1 (Figure 4d) and it interacts with the protein through its coordinating water molecules (Figure 5b).

The overall shape of pU(S)6 is similar to that of pU5 in the MmDXO complex but rather different from that in the CaRai1 complex (Figure 4d). The first two nucleotides of pU(S)6 make conserved interactions with AgRai1 (Figure 5b). The backbone phosphorothioate group of the third nucleotide, U_4_, interacts with Lys254 (motif V) but is about 5 Å away from Arg275 (motif VI). The positions of its ribose and base show clear differences to those of U_4_ in the MmDXO complex, probably because the side chain of Trp154 (αD) in AgRai1 clashes with the position of U_4_ in the MmDXO complex (Figure 4d). This residue is unique to AgRai1 (Supplementary Figure S1). While the five bases of pU5 in the MmDXO complex maintain base stacking, the last two bases of pU(S)6 in the AgRai1 complex are flipped relative to the first four bases, and the last base is positioned against the side chain of Asn253 (αF), at the opening of the active site pocket (Figure 5d).

We also observed a sulfate or phosphate ion (modeled as sulfate) in the active site region of this structure, bound through interactions with the dipole of helix αB (backbone amides of Arg106 and Gly107), the side chain guanidinium group of Arg106 (motif I), and the side chain ammonium ion of Lys198 (β9) (Figure 5b). This sulfate ion is likely located in the general area where the leaving group of the substrate is bound.

AgRai1 has the strongest decapping and exonuclease activities in our assays (Figures 2 and 3). We examined the structure of this complex to identify interactions that may be unique to this enzyme. Three residues were found to be located near pU(S)6 that are present almost exclusively in AgRai1: Lys110 (αB, interacting with the base of U_5_), Trp154 (αD, near U_4_) and Tyr159 (αD, near U_2_ base) (Figure 5c). In addition, we identified Lys198 (β9) that interacts with the sulfate (Figure 5b) as being unique to AgRai1 (Supplementary Figure S1).

### Structure-based mutations affect activity profiles

For both CaRai1 and AgRai1, the mutants were expressed and purified following the same protocol as the wild-type enzyme, and showed the same profile on a gel filtration column (data not shown). The mutants also had essentially the same melting curves as the wild-type enzyme in thermal shift assays (data not shown). These data suggest that the mutations did not disrupt the overall structures of the proteins.

CaRai1 had very low activity in our biochemical assays except for decapping toward unmethylated RNA (Figures 2 and 3, Table 1). The structure of the CaRai1–pU5–Mn^2+^ complex suggested that Glu104 (Figure 4b) may interfere with the binding of the substrate. We therefore created the E104G mutant (Supplementary Figure S1), and observed substantially enhanced decapping activity in our assays (Figure 6a), especially toward the methylated capped substrate (Figure 6b). We also created the E104K mutant, to assess whether the presence of a positive charge could enhance catalytic activity. Our assays showed that this mutant had slightly enhanced activity toward the methylated capped RNA, but was essentially the same as the wild-type enzyme toward the unmethylated capped RNA, suggesting that the bulk of the Lys side chain is detrimental for catalysis. Our structural analysis indicates the increased catalytic activity for the E104G mutant is likely due to enhanced substrate binding, although more efficient catalysis or product release cannot currently be ruled out.

We found that the E104G mutant possessed appreciable 5′-3′ exonuclease activity as well, and was also able to degrade capped RNA and especially 5′ triphosphate RNA (Figure 6c, Supplementary Table S3). Unexpectedly, the E104G mutant also showed activity toward RNA with a 5′ hydroxyl group, albeit weaker, while other Rai1 enzymes and MmDXO (19) did not show this activity at the concentrations tested (10–25 nM). On the other hand, the E104K mutant was relatively inactive toward this panel of substrates, similar to the wild-type enzyme.

To test the importance of the residues unique to AgRai1 that interact with pU(S)6, we created the K110T, Y159E and K198I mutants, changing the residues to their equivalents in another Rai1 protein (Supplementary Figure S1), and replaced Trp154 with Ala (W154A). As discussed earlier, Lys198 likely interacts with the leaving group of the substrate. The W154A and Y159E mutations greatly reduced the decapping activity, while the K110T and K198I mutations had only a small effect (Figure 6d, e). We also created the W154A/Y159E double mutant and the W154A/Y159E/K110T triple mutant, and found that they had essentially no decapping activity. The mutants also had greatly reduced 5′-3′ exonuclease activity (Figure 6c).

The W154A mutation is unlikely to affect the binding mode of the first two nucleotides of the product (U_2_ and U_3_), but could affect that of the rest of the RNA body. The large effect of this mutation suggests that the binding mode of the RNA body could impact the catalytic activity of Rai1, which could have wider implications given the differences in the binding modes of this region of the substrates among the Rai1 homologs (Figure 4d). The exact mechanism for this effect will need to await additional information from further studies, especially the binding modes of substrates to these enzymes.

Overall, our studies have revealed remarkable variability in the activity profiles of the fungal Rai1 homologs, despite their overall amino acid sequence conservation and structural similarity. While the binding modes of the first two nucleotides of the RNA body are generally conserved among the enzymes, the rest of the RNA can assume rather diverse structures. Mutations of residues that contact the RNA but are not conserved among the Rai1 homologs can have dramatic effects on the activity profiles, indicating their functional importance and suggesting that these residues help define the different catalytic activities of these enzymes.

We also observed a new catalytic activity for Rai1 homologs, in that AgRai1, CgRai1 and SsRai1 release GTP (pppG) from 5′ triphosphate RNA. This 5′ triphosphonucleotide hydrolase (TPH) activity is distinct from that of the canonical 5′-3′ exonucleases, which are active toward 5′ monophosphate RNA but are inactive toward 5′ triphosphate RNA. Currently we do not understand why AgRai1, CgRai1 and SsRai1 can accommodate GTP as the leaving group, while SpRai1 uses PPi as the leaving group. Having structures of these enzymes in complex with the substrate will be important in revealing the mechanism(s) of these different activity profiles. Interestingly, our earlier studies with the H163G and H163G/D167K mutants of KlDxo1 also showed TPH activity (18). In addition, the E104G mutant of CaRai1 has acquired appreciable activity toward 5′ hydroxyl RNA, again indicating the remarkable diversity in the catalytic activity of the Rai1 enzymes.

## ACCESSION NUMBERS

The atomic coordinates have been deposited at the Protein Data Bank, with accession codes 5BTB, 5BTH, 5BTO, 5BTE and 5BUD.

## SUPPLEMENTARY DATA

Supplementary Data are available at NAR Online.

SUPPLEMENTARY DATA
