# Supplementary material for: Structural and biochemical studies of the distinct activity profiles of Rai1 enzymes
Source: Nucleic Acids Res. 2015 Jun 22;43(13):6596–606. doi: 10.1093/nar/gkv620 (PMC4513879; doi:10.1093/nar/gkv620)
Supplement: SUPPLEMENTARY DATA [file supp_43_13_6596__index.html]

Structural and biochemical studies of the distinct activity profiles of Rai1 enzymes — Structural and biochemical studies of the distinct activity profiles of Rai1 enzymes — Structural and biochemical studies of the distinct activity profiles of Rai1 enzymes — SUPPLEMENTARY DATA 

# Structural and biochemical studies of the distinct activity profiles of Rai1 enzymes

## SUPPLEMENTARY DATA

- SUPPLEMENTARY DATA
