# Supplementary material for: Structural and biochemical studies of the distinct activity profiles of Rai1 enzymes
Source: Nucleic Acids Res. 2015 Jun 22;43(13):6596–606. doi: 10.1093/nar/gkv620 (PMC4513879; doi:10.1093/nar/gkv620)
Supplement: SUPPLEMENTARY DATA [file supp_gkv620_nar-01078-v-2015-File008.pdf]

Supplementary Table 1: Quantitation of Fig. 3A

Rai1 Exonuclease Activities

	Time (min)	**m^7GpppRNA**	% RNA remaining	**GpppRNA**	% RNA remaining	**pppRNA**	% RNA remaining	**pRNA**	% RNA remaining
**AgRai1**	0	68348	100%	77240	100%	82903	100%	78637	100%
	20	23972	35%	34933	45%	35682	43%	34672	44%
	40	11910	17%	20741	27%	19292	23%	18451	23%
**CaRai1**	0	60647	100%	69637	100%	74044	100%	71644	100%
	20	62468	103%	69849	100%	72002	97%	68659	96%
	40	56275	93%	67564	97%	73880	100%	68944	96%
**CgRai1**	0	63809	100%	69298	100%	74902	100%	68120	100%
	20	63034	99%	64899	94%	71431	95%	67702	99%
	40	61359	96%	64682	93%	70740	94%	66891	98%
**LtRai1**	0	63738	100%	66623	100%	71012	100%	68254	100%
	20	50469	79%	57225	86%	61844	87%	58470	86%
	40	42093	66%	56179	84%	52841	74%	56177	82%
**ScRai1**	0	69709	100%	72589	100%	73853	100%	72406	100%
	20	70683	101%	75603	104%	74429	101%	69762	96%
	40	69482	100%	74206	102%	70063	95%	68462	95%
**SpRai1**	0	72493	100%	72941	100%	71030	100%	71240	100%
	20	72992	101%	71838	98%	67624	95%	71592	100%
	40	66817	92%	70880	97%	66296	93%	70131	98%
**SsRai1**	0	71487	100%	74401	100%	68885	100%	68795	100%
	20	51645	72%	57196	77%	49532	72%	43036	63%
	40	38077	53%	51153	69%	47862	69%	39374	57%
**VpRai1**	0	65864	100%	78180	100%	71742	100%	71122	100%
	20	71732	109%	74808	96%	71120	99%	68907	97%
	40	67409	102%	71853	92%	71529	100%	73001	103%

Supplementary Table 2. Quantitation of Fig. 3C

Rai1 Exonuclease Activities

	Time (min)	**m^7GpppRNA**	% RNA remaining	**GpppRNA**	% RNA remaining	**pppRNA**	% RNA remaining	**pRNA**	% RNA remaining
**AgRai1**	0	122962	100%	88111	100%	102052	100%	95578	100%
**Wt**	20	72184	59%	67189	76%	72171	71%	82030	86%
	40	35363	29%	37606	43%	38607	38%	19502	20%
**AgRai1**	0	101183	100%	92549	100%	93272	100%	95434	100%
**E215A/D217A**	20	102241	101%	87880	95%	88209	95%	87016	91%
	40	87421	86%	82528	89%	92698	99%	85974	90%

Supplementary Table 3: Quantitation of Fig. 6C

Rai1 and mutants Exonuclease Activities

	Time (min)	**m^7GpppRNA**	% RNA remaining	**pppRNA**	% RNA remaining	**pRNA**	% RNA remaining	**OH-RNA**	% RNA remaining
**AgRai1**	0	103507	100%	92192	100%	88274	100%	88271	100%
	20	60724	59%	63127	68%	63799	72%	84584	96%
	40	52946	51%	59902	65%	57515	65%	81888	93%
**AgRai1 K198I**	0	86674	100%	82324	100%	77426	100%	82781	100%
	20	64381	74%	72817	88%	54253	70%	82327	99%
	40	41985	48%	66810	81%	40939	53%	83528	101%
**AgRai1 E154A**	0	84053	100%	78259	100%	82522	100%	84525	100%
	20	61048	73%	73217	94%	60193	73%	86228	102%
	40	55432	66%	68091	87%	57294	69%	86554	102%
**AgRai1 Y159E**	0	87361	100%	77335	100%	80672	100%	85425	100%
	20	85170	97%	75597	98%	80936	100%	85156	100%
	40	90459	104%	73949	96%	81561	101%	89198	104%
**AgRai1 W154A/Y159E**	0	88925	100%	76338	100%	85540	100%	76028	100%
	20	81965	92%	79924	105%	85083	99%	77384	102%
	40	69236	78%	78811	103%	79157	93%	75545	99%
**AgRai1 K110T**	0	81278	100%	80404	100%	79451	100%	82541	100%
	20	69641	86%	71844	89%	69470	87%	79103	96%
	40	66005	81%	68281	85%	61978	78%	80435	97%
**AgRai1 E154A/Y159E K110T**	0	83542	100%	76464	100%	74862	100%	76536	100%
	20	85104	102%	74044	97%	80142	107%	76864	100%
	40	85463	102%	79713	104%	80732	108%	77294	101%
**CaRai1**	0	83938	100%	86034	100%	92708	100%	92021	100%
	20	79772	95%	83164	97%	89668	97%	93115	101%
	40	78265	93%	81200	94%	87851	95%	88019	96%
**CaRai1 E104G**	0	109712	100%	97499	100%	79254	100%	97800	100%
	20	69677	64%	46699	48%	57724	73%	63986	65%
	40	68950	63%	34576	35%	53184	67%	63392	65%
**CaRai1 E104K**	0	80228	100%	84780	100%	91567	100%	85761	100%
	20	77300	96%	69028	81%	90382	99%	84840	99%
	40	68300	85%	68953	81%	85595	93%	79171	92%

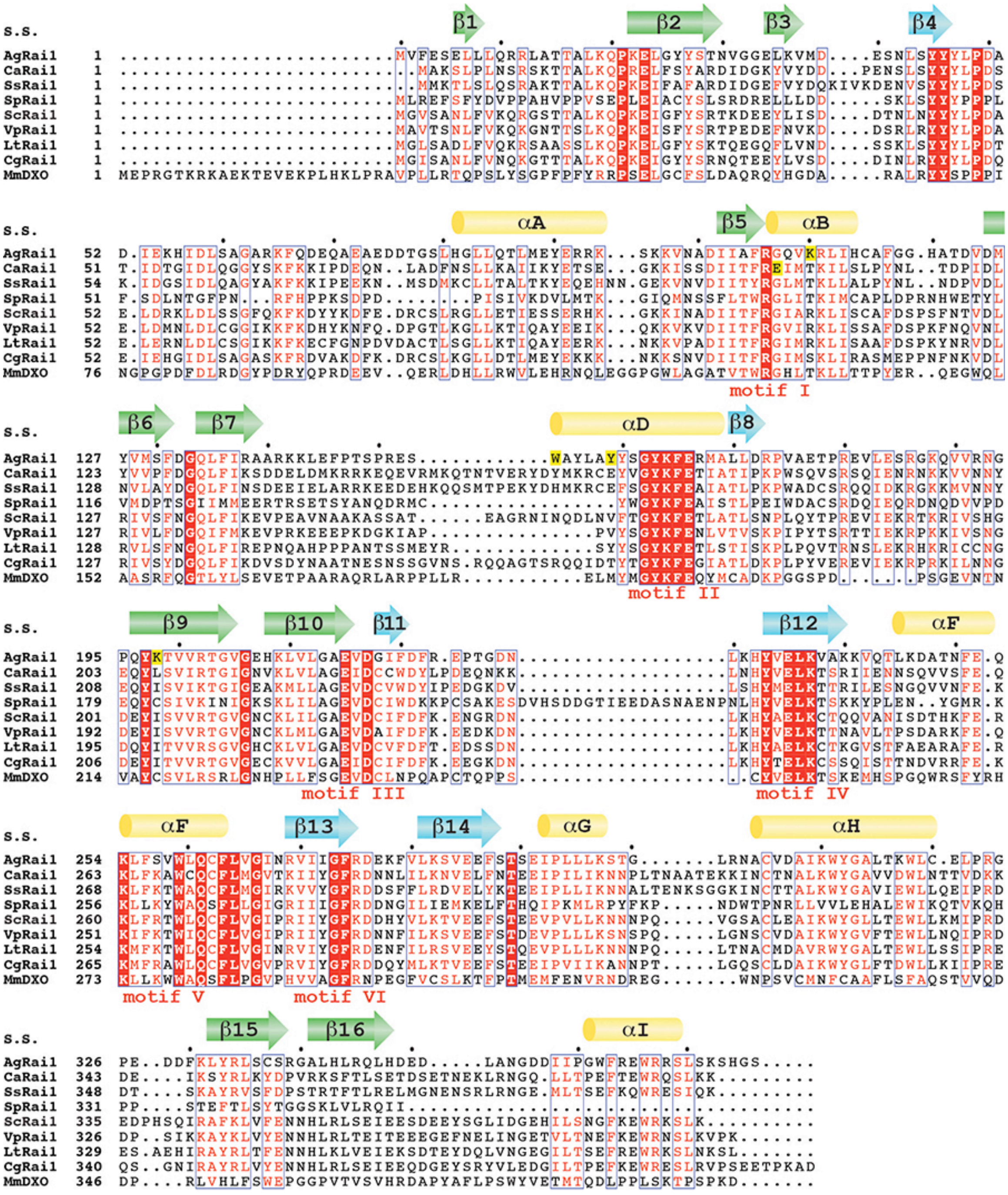

Supplementary Fig. 1. Sequence alignment of fungal Rai1 homologs and mouse DXO (MmDXO). The secondary structure elements in the AgRai1 structure are shown (S. S.), and the six conserved sequence motifs are labeled. Strictly conserved residues among the aligned sequences are highlighted in red, mostly conserved residues in red. Residues that were selected for mutagenesis studies in AgRai1 and CaRai1 are highlighted in yellow.

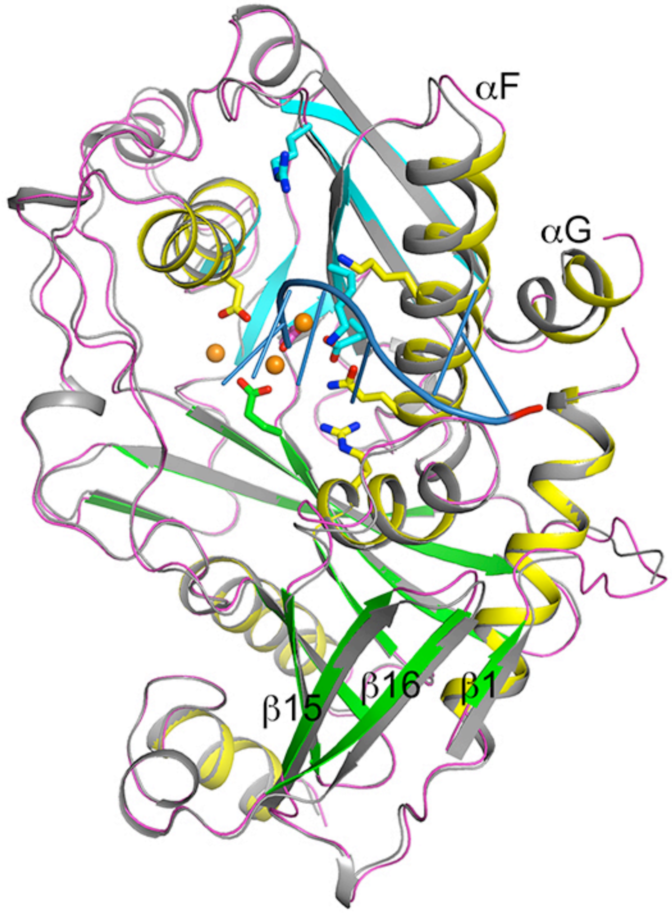

Supplementary Fig. 2. Overlay of the structure of AgRai1 in a ternary complex with pU(S)6 and Mn^{2+} (in color) with that of free AgRai1 (gray).

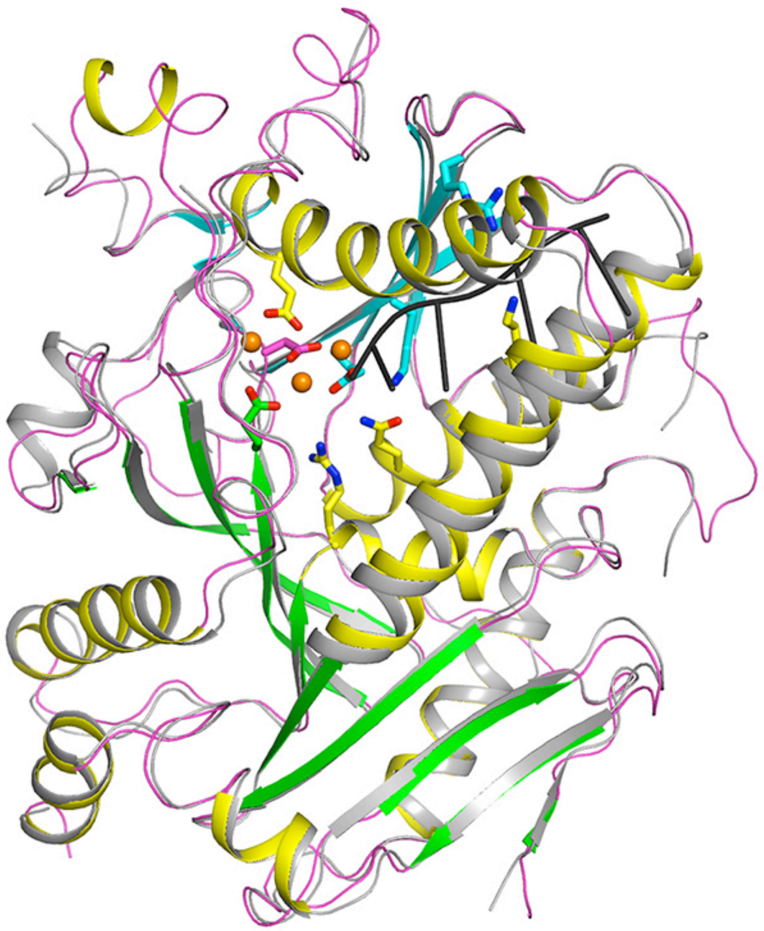

Supplementary Fig. 3. Overlay of the structure of CaRai1 in a ternary complex with pU5 and Mn^{2+} (in color) with that of SsRai1 (gray).

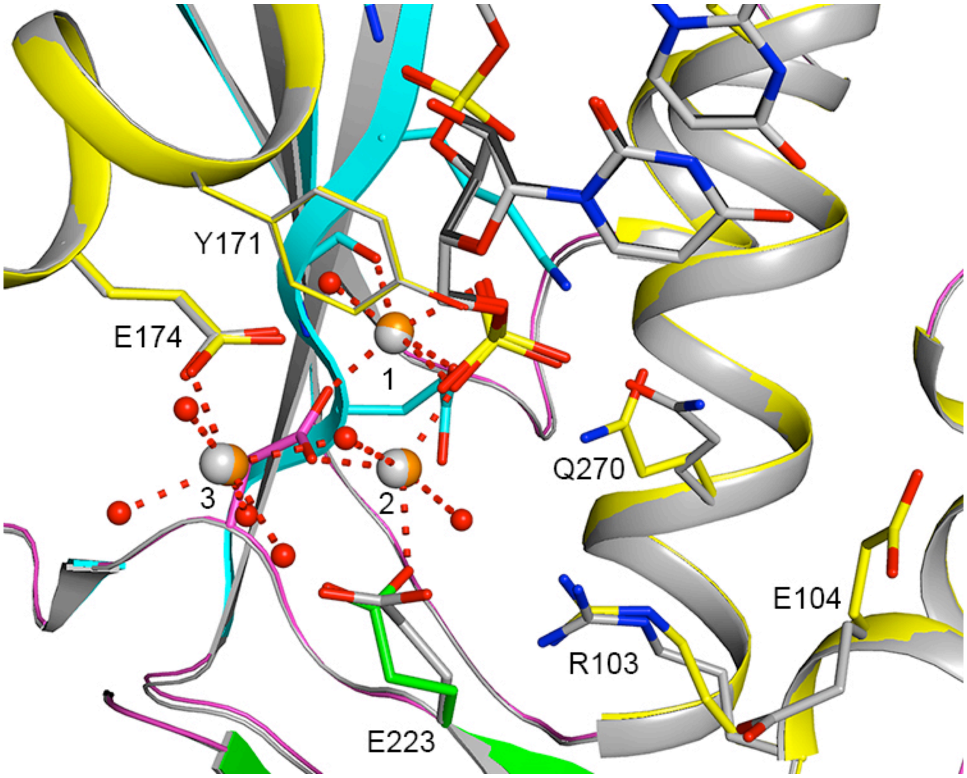

Supplementary Fig. 4. Overlay of the active site region of the two molecules of CaRai1 in the crystallographic asymmetric unit of the ternary complex with pU5 and Mn^{2+}.
